# Minimizing Task Age upon Decision for Low-Latency MEC Networks Task Offloading with Action-Masked Deep Reinforcement Learning

**DOI:** 10.3390/s24092812

**Published:** 2024-04-28

**Authors:** Zhouxi Jiang, Jianfeng Yang, Xun Gao

**Affiliations:** Electronic Information School, Wuhan University, Wuhan 430072, China; jiangzhouxi@whu.edu.cn (Z.J.); gaoxun@whu.edu.cn (X.G.)

**Keywords:** low-latency mobile edge computing, age upon decision, finite blocklength regime, deep reinforcement learning, maskable proximal policy optimization

## Abstract

In this paper, we consider a low-latency Mobile Edge Computing (MEC) network where multiple User Equipment (UE) wirelessly reports to a decision-making edge server. At the same time, the transmissions are operated with Finite Blocklength (FBL) codes to achieve low-latency transmission. We introduce the task of Age upon Decision (AuD) aimed at the timeliness of tasks used for decision-making, which highlights the timeliness of the information at decision-making moments. For the case in which dynamic task generation and random fading channels are considered, we provide a task AuD minimization design by jointly selecting UE and allocating blocklength. In particular, to solve the task AuD minimization problem, we transform the optimization problem to a Markov Decision Process problem and propose an Error Probability-Controlled Action-Masked Proximal Policy Optimization (EMPPO) algorithm. Via simulation, we show that the proposed design achieves a lower AuD than baseline methods across various network conditions, especially in scenarios with significant channel Signal-to-Noise Ratio (SNR) differences and low average SNR, which shows the robustness of EMPPO and its potential for real-time applications.

## 1. Introduction

Mobile Edge Computing (MEC) has been recognized as a key enabling technology supporting ultra-reliable and latency-sensitive applications in the future Sixth-Generation Mobile Networks (6G) [1,2], such as autonomous driving [3], simultaneous localization and mapping [4], intelligent manufacturing [5], and unmanned aerial vehicle [6]. For instance, in autonomous driving, vehicles need to promptly transmit and process real-time data from various sources, including sensors on vehicles and other roadside servers, to achieve comprehensive awareness of the road environment and make reliable decisions for the following actions. Since vehicles may travel at high speeds, the process needs to be completed within tens of milliseconds and with high reliability. Considering the limited performance and resources of User Equipment (UE), and dynamic wireless channels, Ultra-Reliable Low-Latency Communications (URLLC) in MEC networks is challenging.

In the MEC network, an edge server typically serves multiple UEs. Given the finite communication and computation resources, there is often competition among UEs. Therefore, offloading decisions and resource allocation impact system performance and should be taken into account during system design [7,8,9,10,11,12]. In [7,8], the authors optimize latency in the MEC network by controlling the offloading strategy and power allocation. In addition to making an offloading decision, the authors in [9] allocate the CPU frequency of the MEC server and assign wireless bandwidth for the transmission. The work in [10] provides an optimal design to minimize the overall error probability by server selection and time allocation. The authors of [11] introduce a fast futures-enabled resource trading mechanism to determine optimal transmission power and further propose a hybrid market approach that integrates futures and spot trading to facilitate resource trading in [12].

However, all of these studies are based on the assumption of infinite blocklength, which means the transmission of data packets is considered arbitrarily reliable at Shannon’s capacity. To meet low latency requirements, data packets wirelessly uploading/offloading in MEC networks are more likely to operate via short block codes, so-called communications in the Finite Blocklength (FBL) regime [13]. In the FBL regime, transmission error possibly occurs even when the coding rate is set to be lower than the Shannon capacity. When transmitting data packets with shorter blocklength to reduce transmission delay, the probability of transmission error increases correspondingly. Therefore, minimizing latency with a reliability guarantee under the FBL regime should be carefully investigated.

In the past few years, a set of works have taken the FBL impacts into consideration in the performance analysis and system design for MEC networks [14,15,16,17]. The study in [14] introduces an energy-efficient algorithm for dynamic computation offloading in a multi-access edge computing scenario, focusing on delay-critical applications by incorporating URLLC through FBL and reliability constraints to manage radio and computational resources jointly. The authors of [15] propose an efficient algorithm for an unmanned aerial vehicle-enabled MEC system with URLLC-based offloading to minimize the maximum computation latency, considering the FBL transmission and its impact on the data rate. Moreover, the work in [16] proposes an MEC-aided integrated sensing and communication scheme that leverages short-packet transmissions to efficiently offload radar-sensing data to an edge-server, ensuring low latency and high reliability for radar data analysis while minimizing system energy consumption. In [17], the authors investigate the effects of short-packet transmission on the radio resource allocation and minimize the energy cost for mission-critical Internet-of-Things (IoT) in an MEC system. Some authors have also combined FBL with various communication technologies, such as non-orthogonal multiple access and retransmission, to analyze the impact on system performance [18,19,20,21]. The authors in [18] provide a design that maximizes the effective capacity via FBL transmission. In [19], a joint optimization problem is formulated by determining the user grouping and allocating blocklength. In [20], the authors propose energy-efficient retransmission schemes for MEC networks with a hybrid automatic repeat request, optimizing the number of retries and transmission parameters under FBL constraints to minimize energy consumption for latency-critical tasks. In [21], the authors allocate blocklength to improve the average end-to-end reliability through a deep reinforcement learning approach. However, existing studies often focus on the transmission delay under the FBL regime. They tend to overlook the queuing delay, especially when tasks involve multiple related data packets.

Since transmission delay does not fully capture the total time taken for transmitting packets, especially ignoring the waiting time of packets at the source, some researchers have introduced the Age of Information (AoI) [22]. The AoI concept, which includes the waiting time of packets at the source, offers a more accurate assessment of packet freshness.

Recently, a set of studies have analyzed the AoI performance of networks operating with FBL codes. The authors in [23,24] present a novel study for downlink cellular networks and orthogonal frequency division multiple access systems to minimize AoI, considering the impact of FBL. In [25], authors optimize the tail distribution of AoI in vehicular networks by employing extreme value theory. The work in [26] investigates the correlation and joint optimization of AoI and peak AoI in a last-come first-served system with retransmission and non-preemption policies. Some other recent studies have analyzed the AoI within the MEC networks. In [27], the authors investigate the average AoI in a wireless-powered MEC system and derive a closed-form expression for it. In [28], the problem of minimizing AoI in an MEC system for computation-intensive status updates is addressed with the no-wait policy and interval-wait policy. The authors of [29] present an AoI-based optimization strategy for computation offloading and transmission scheduling in MEC-enabled IoT networks. In [30], the authors analyze the average AoI and the average peak AoI in a multi-user MEC system, where a base station transmits computation-intensive packets to UEs. However, in some scenarios, data packets are used for decision-making after transmission. The freshness of the data at the time of decision is more critical than at the moment transmission is completed. The AoI does not capture the packets’ freshness for decision-making purposes.

More recently, the concept of Age upon Decision (AuD) has been introduced in [31]. The AuD measures how old the information is when it is used to make a decision, whereas AoI measures the information received. The authors investigate the impact of scheduling update arrival and decision-making processes on the average AuD in [32,33]. Then, the authors of  [34] explored the AuD in IoT-based wireless networks with truncated hybrid automatic repeat requests, focusing on the impact of FBL and multiple sources. Although much of the research has looked at the AuD for data packets, the task AuD that involves multiple data packets still requires further investigation. To the best of our knowledge, there is also a missing analysis and optimization of a task’s AuD with multiple related data packets in low-latency MEC networks under the FBL regime.

In this paper, we consider task transmission and computation in a low-latency MEC network with multiple UEs and one MEC edge server. To minimize the average task AuD, we allocate finite blocklength resources to UEs and decide the transmission moment. We propose an action-masked Proximal Policy Optimization (PPO) algorithm that adapts to time-varying independent channels and randomly arriving tasks while ensuring reliable task transmission. Our main contributions are summarized as follows:

- We introduce a low-latency MEC network where tasks are composed of multiple data packets transmitted by various UEs through wireless channels to an edge server under the FBL regime. We analyze the impact of blocklength allocation and UE selection on the error probability of data packet transmission and the task AuD.
- We formulate an optimization problem to minimize the average task AuD by jointly allocating blocklength and selecting UEs while ensuring error probability constraints. Subsequently, we transform this into a UE selection problem that adheres to the error probability constraints dictated by the FBL regime.
- We propose an Error Probability-Controlled Action-Masked Proximal Policy Optimization (EMPPO) algorithm considering dynamic task generation and random fading channels. By masking actions that cannot satisfy error probability constraints, our algorithm prevents the exploration of ineffective actions, thereby enhancing efficiency.
- Simulation results are provided to validate the performance of our proposed method compared to server baseline methods, especially the standard DRL methods. We demonstrate the robustness of our method under various network conditions, such as varying task arrival probabilities, channel correlation coefficients, and Signal-to-Noise Ratio (SNR).

The rest of the paper is organized as follows: In Section 2, we describe the low-latency MEC system model and FBL performance and task AuD. We state the optimization problem of minimizing the task AuD and present an action-masked DRL algorithm in Section 3. Then, we evaluate the performance and show the simulation results in Section 4. Finally, we conclude our work in Section 5.

## 2. Preliminaries

In this section, we introduce the system model of the low-latency MEC network and FBL regime in detail. Then, we introduce and analyze the task AuD in a two UEs low-latency MEC scenario.

### 2.1. System Model

**Figure 1:**
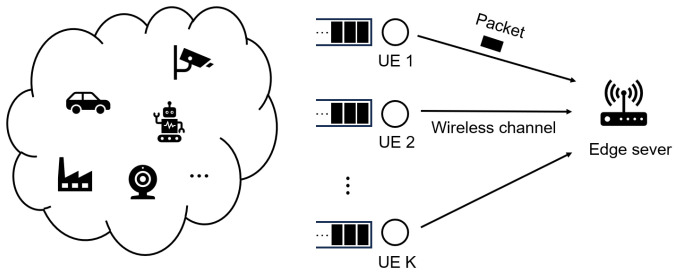
System model of low-latency MEC network.

As shown in Figure 1, the system model of the low-latency MEC network we consider is composed of a set of UEs $U=\left\{1,2,\cdots ,K\right\}$ and one MEC server. We discretize time into frames of consistent length. At the beginning of each frame, the task arrives randomly. A task consists of multiple data packets of different sizes. Each packet is stored in a user’s queue and then transmitted to the edge server at a later frame. One frame may transmit one or more packets. Once all data packets of a task have been successfully transmitted, the task can start computation and then be used to decide when the computation is finished.

Specifically, we discretize time into a set of time frames $T=\left\{1,2,\cdots ,T\right\}$. Each frame is comprised of *n* symbols, which are the smallest units required to represent the transmitted data in our communication system. A symbol is not merely a bit, instead, it encapsulates a discrete value or a set of bits according to the modulation scheme used. The duration of each symbol is $T_{s}$, which is how long it takes to transmit one symbol. Therefore, the total duration of the frame $T_{f}$ can be represented as

(1) $$T_{f}=nT_{s}$$

**Figure 2:**
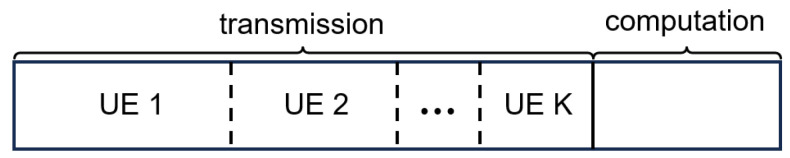
Frame structure.

Each frame can be split into a communication phase and a computation phase. The frame structure is shown in Figure 2. During the transmission phase, data packets from different UEs are transmitted to the edge server via the wireless channels using time-division multiple access. During the computation phase, we consider joint-packet task scenarios. That is, a task is made up of data packets from multiple UEs. A task’s computation and decision-making process can only be initiated when all data packets involved have been transmitted (transmission can be distributed across different frames). Considering that the ability to start decision-making for a task depends mainly on whether the last data packet of the task has been successfully transmitted, we utilize a fixed time length for the computation phase. Tasks in which all packets have been fully transmitted can proceed with calculation and decision-making in the computation phase.

Data packets may arrive at the beginning of each frame, that is, before the transmission starts. A data packet with packet size $m_{k,t}$ (in bits) arrives at the UE *k* in frame *t* with a probability of *p*, suggesting that the data packet arrivals conform to an On/Off Markov arrival model:

(2) $$P=\left\{\begin{array}{cc} p & \mathrm{packet}\;\mathrm{arrives}\\ 1-p & \mathrm{packet}\;\mathrm{does}\;\mathrm{not}\;\mathrm{arrive} \end{array}\right.$$

Furthermore, for each UE, a data packet is then cached in an independent First-In-First-Out (FIFO) queue.

Notably, we consider joint task decisions that require multiple data packets. This suggests that for a specific task, its data packets always arrive at various UEs at the same time (e.g., in an autonomous driving scenario, we can realize a comprehensive perception of the road environment at a specific moment by aggregating the data captured from different sensors at the exact moment).

Additionally, due to potential variations in the types of UEs, the sizes of data packets arriving at each UE may differ. We assume that the packet size $m_{k,t}$ is uniformly distributed across the range [a,b], ensuring that each packet size within this interval is equally likely to occur. The probability density function of the packet size $m_{k,t}$ is given by:

(3) $$f\left(m_{k,t}\right)=\left\{\begin{array}{cc} \frac{1}{b-a} & a\leq m_{k,t}\leq b\\ 0 & \mathrm{otherwise} \end{array}\right.$$

Channels are considered to experience quasi-static Rayleigh fading. Therefore, as the duration of a frame $T_{f}$ is shorter than the channel coherent time, the channel fading remains constant within the frame but may vary between the current frame and the next. Channels between different UE and edge servers are considered to be independent.

Thus, for channel state $h_{k,t}$, we adopt the widely used Jakes model to depict the correlation of channel states between frames:

(4) $$h_{k,t}=\rho h_{k,t-1}+\sqrt{1-\rho^{2}}\Delta h_{k,t}$$

where $h_{k,t}$ is the channel state of frame *t* between user *k* and the edge server, $h_{k,t-1}$ is the channel state of frame t−1 between user *k* and the edge server, ρ∈[0,1] is the channel correlation coefficient, and $\Delta h_{k,t}∼CN\left(0,1\right)$ is a complex Gaussian random variable. The channel state $h_{k,t}$ is modeled as influenced by the previous state $h_{k,t-1}$ through the correlation coefficient ρ and a random component $\sqrt{1-\rho^{2}}\Delta h_{k,t}$, which represents the time correlation and randomness of the wireless channel. Hence, we have the received signal-to-noise (SNR) given by

(5) $$\gamma_{k,t}={\bar{\gamma }}_{k}h_{k,t}^{2}$$

where ${\bar{\gamma }}_{k}$ is the average SNR of channel *k*. We consider instantaneous perfect Channel State Information (CSI) is available.

### 2.2. FBL Communication Performance

To achieve low-latency transmission, the data packet is transmitted within the FBL regime, which means the FBL regime should be considered during the transmission phase.

Following the FBL regime [13], the coding rate $r_{k,t}$ for an additive white Gaussian noise channel between UE *k* and edge server in frame *t* is shown to have the following approximation:

(6) $$r_{k,t}=R\left(\gamma_{k,t},\varepsilon_{k,t},n_{k,t}\right)\approx C\left(\gamma_{k,t}\right)-\sqrt{\frac{V(\gamma_{k,t})}{n_{k,t}}}Q^{-1}\left(\varepsilon_{k,t}\right)$$

where $\gamma_{k,t}$ is the instantaneous SNR, $\varepsilon_{k,t}$ is the error probability of transmission, $n_{k,t}$ is the blocklength, $C\left(\gamma_{k,t}\right)=\log_{2}\left(1+\gamma_{k,t}\right)$ is the Shannon capacity, $V\left(\gamma_{k,t}\right)=1-\frac{1}{{\left(1+\gamma_{k,t}\right)}^{2}}$ is the channel dispersion, $Q\left(x\right)=\int_{x}^{\infty }\frac{1}{\sqrt{2\pi }}e^{\frac{-t^{2}}{2}}dt$ is the Gaussian Q-function, and $Q^{-1}\left(x\right)$ is its inverse function. From (6), the error probability of the transmission $\varepsilon_{k,i}$ can be expressed as:

(7) $$\varepsilon_{k,t}\approx Q\left(\frac{C\left(\gamma_{k,t}\right)-r_{k,t}}{\sqrt{\frac{V(\gamma_{k,t})}{n_{k,t}}}}\right)$$

For a data packet with packet size $m_{k,t}$ transmitted at the UE *k* in frame *t*, the coding rate $r_{k,t}$ can also be expressed as:

(8) $$r_{k,t}=\frac{m_{k,t}}{n_{k,t}}$$

From the analysis above, we know that allocating more blocklength for the transmission of a data packet will reduce the transmission error probability. However, it will also result in longer transmission times. Additionally, the error probability is affected by the time-varying CSI. Therefore, choosing the appropriate blocklength and transmission timing for data packets is important to achieve a trade-off between error probability and transmission time.

### 2.3. Task AuD

**Figure 3:**
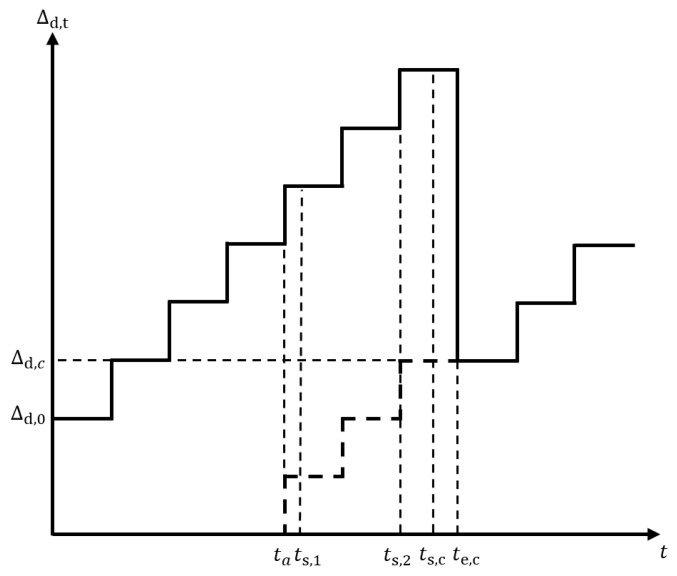
Task AuD of the low-latency MEC network.

To better understand the task AuD, we use a simple example comprising two UEs and an edge server for low-latency MEC network. Figure 3 shows the task AuD of the network.

As shown in Figure 3, the system’s initial AuD is $\Delta_{d,0}$. As time is divided into frames, if no task completion decision is made, the AuD for the next frame will be

(9) $$\Delta_{d,1}=\Delta_{d,0}+T_{f}$$

Then, a task arrives at the UEs at $t_{a}$. This task consists of two packets $m_{1}$ and $m_{2}$, which are individually cached in the queues of UE1 and UE2. After waiting in the queue, the packets at both UE1 and UE2 start transmitting to the edge server (may transmit in different frames). Specifically, the packet at UE1 starts transmission at $t_{s,1}$ and the packet at UE2 begins transmission at $t_{s,2}$. According to the system model, packets must be completed within the frame when starting transmission. After the two packets have completed their transmission (i.e., the task transmission is completed), the task begins to compute on the edge server at a subsequent moment, represented as $t_{s,c}$, and the computation ends at $t_{e,c}$. As shown in Figure 2, the computation phase of the task consumes a fixed time length. The computation finishes at the end of the frame with making the task decision. Since the task is decided, we have the new task AuD of the network in the current frame:

(10) $$\Delta_{d,c}=t_{e,c}-t_{a}$$

Equation (10) shows that the new task AuD is determined by the time that all data packets of the task complete transmission and the task completes computation. Thus, for the task AuD $\Delta_{d,t}$ in frame *t*, the task AuD $\Delta_{d,t+1}$ in the next frame is determined by:

(11) $$\Delta_{d,t+1}=\left\{\begin{array}{cc} \Delta_{d,t}+T_{f} & \mathrm{if}\;\mathrm{no}\;\mathrm{task}\;\mathrm{decided}\;\mathrm{in}\;\mathrm{frame}\;t\\ t_{e,c}-t_{a} & \mathrm{if}\;\mathrm{the}\;\mathrm{task}\;\mathrm{decided}\;\mathrm{in}\;\mathrm{frame}\;t\; \end{array}\right.$$

where $T_{f}$ is the frame duration, $t_{e,c}$ is the timestamp when the task computation is completed and the decision is made, $t_{a}$ is the timestamp when the task arrives at the UEs.

Therefore, for an MEC network that has gone through *N* frames, its average task AuD is defined as:

(12) $${\bar{\Delta }}_{d}=\frac{1}{N}\sum_{t=1}^{N}\Delta_{d,t}$$

From the example, it can be seen that, compared to the AuD of a single data packet, which only depends on when the packet is used for decision-making after being transmitted to the edge server, Task AuD requires that all packets of a task reach the edge server before they can participate in decision-making together. The Task AuD of a task is largely influenced by the packet that arrives last at the edge server.

## 3. DRL-Based Joint User Selection and Blocklength Allocation Design

In this section, we formulate the optimization problem of UE selection and blocklength allocation. Then, we formulate the task offloading problem as a Markov Decision Process (MDP) that can be solved by deep reinforcement learning methods. Finally, we investigate the EMPPO algorithm in detail.

### 3.1. Problem Formulation

For a network composed of *K* UEs and one MEC server, we will decide which packets will be transmitted, and the blocklength to be used for each packet in each frame to minimize long-term average task AuD. Considering that the data packets on each UE are cached in a FIFO queue, we assume each UE can transmit at most one packet within a frame. Therefore, we can use a set to represent the selection of UEs and blocklength allocation in frame *t*:

(13) $$N_{t}=\left\{n_{1,t},n_{2,t},\cdots ,n_{k,t}\right\},n_{k,t}\in N$$

where $n_{k,t}$ is the blocklength assigned to UE *k* in frame *t*. $n_{k,t}=0$ means that the UE does not transmit packets in this frame. From (13), the optimization problem of task offloading can be formulated as follows:

(14a) $$\begin{array}{cc} \min_{N_{i},t\in T}\; & \bar{\Delta_{d}} \end{array}$$

(14b) $$\begin{array}{cc} s.t.\; & \sum_{k=1}^{K}n_{k,t}=n_{c},\forall i\in T\;n_{k,t}\in N \end{array}$$

(14c) $$\begin{array}{cc} \; & \varepsilon_{k,t}\leq \varepsilon_{max},\forall k\in U,t\in T \end{array}$$

$\bar{\Delta_{d}}$ is the optimization objective, defined as minimizing the average AuD in Equation (12). The constraint (14b) guarantees that the total blocklength equals the available blocklength for the transmission phase in a frame. The constraint (14c) ensures that the error probability for each transmitted packet does not exceed the error probability constraint $\varepsilon_{max}$.

From (7) and (8), it is evident that the error probability monotonically decreases as the blocklength increases. Thus, for a given error probability constraint $\varepsilon_{max}$, with the known SNR $\gamma_{k,t}$ and data packet size $m_{k,t}$, we can determine the optimal blocklength $n_{o,k,t}$ for transmission such that the packets are transmitted exactly meeting the error probability constraint.

Therefore, when we select a UE, the transmitted blocklength is determined uniquely. By using the binary decision variables, we can transform the joint problem of UE selection and blocklength allocation into a UE selection problem. At frame *t*, the set of binary decision variables used to determine whether the UE transmits is:

(15) $$X_{t}=\left\{x_{1,t},x_{2,t},\cdots ,x_{K,t}\right\},x_{K,t}\in \left\{0,1\right\}$$

Then, we can transform the optimization problem into:

(16a) $$\begin{array}{cc} \min_{X_{t},t\in T}\; & \bar{\Delta_{d}} \end{array}$$

(16b) $$\begin{array}{cc} s.t.\; & \sum_{k=1}^{K}n_{o,k,t}\leq n_{c},\forall t\in T \end{array}$$

(16c) $$\begin{array}{cc} \; & X_{t}=\left\{x_{1,t},x_{2,t},\cdots ,x_{K,t}\right\},x_{K,t}\in \left\{0,1\right\} \end{array}$$

Constraint (16b) is the total blocklength constraint for a frame. Constraint (16c) is the set of binary decision variables, where $x_{K,t}$ represents the binary decision for each UE *K* at frame *t*, taking a value of either 0 or 1. $x_{K,t}=1$ means UE *K* transmits its data packet using the optimal blocklength that precisely meets the error probability constraint in frame *t*, whereas $x_{K,t}=0$ means that the UE *K* will not transmit a data packet in that frame. Given that the selected packets for transmission are transmitted in such a way to exactly meet the error rate constraint, the total blocklength may not exactly equal the blocklength available for transmission in one frame. In real-world scenarios, we allocate the remaining blocklength to the packets to achieve lower transmission error probability.

### 3.2. MDP Formulation

UE selection problem (16a) can be considered as a sequential decision-making process. At any given frame, we can only access the present and historical states, and make decisions under the current state which will influence future states and decisions. Therefore, obtaining the optimal global solution for this problem is impossible. However, it can be modeled as a MDP and solved by reinforcement learning methods.

Reinforcement Learning tasks are usually modeled by MDP. Each state of the Reinforcement Learning system must have the Markov property, meaning once the current system state is known, future states are solely dependent on it and not on past states. An MDP in Reinforcement Learning can be described with the tuple (S,A,P,R,γ). *S* represents the state space, which is the set of all possible states of the environment. *A* is the action space representing the set of all possible actions the agent can take. *P* is the set of state transition probabilities when the agent performs an action in a given state. *R* is the reward function. γ is the discount factor that dictates the degree to which future rewards brought by actions are discounted when calculating cumulative rewards.

The classical reinforcement learning process includes the following steps:

(1) At every time step *t*, the agent observes the current environment state $s_{t}\in S$.
(2) The agent selects an action $a_{t}$ according to the policy $\pi (s_{t})$ based on the current environmental state.
(3) The environment transitions to state $s_{t+1}$ due to action $a_{t}$ and gives a reward $r_{t}$ to the agent.

The learning process of reinforcement learning is an interactive process between the agent and the environment. It allows the agent to explore in an unfamiliar environment where different actions lead to different rewards. As the agent interacts continuously with the environment, it constantly improves its strategy based on changes in rewards and environmental states. After many trials and errors, it obtains the strategy with the most cumulative rewards.

The agent continuously updates and iterates its strategy according to the rewards obtained from the actions taken in different environmental states in the ongoing interactive process, aiming to obtain as much cumulative reward as possible.

In our low-latency MEC scenario, the definitions of state space, action space, and reward function are as follows.

- State Space: The state space is composed of three components, which are the age and size of the oldest data packet in each UE’s queue (the oldest data packet will be at the head of the queue), and the real-time SNR of the channels. Therefore, the state in frame *i* can be represented as:
  (17) $$s_{t}=\left\{\Delta_{1,t},\Delta_{2,t},\cdots ,\Delta_{K,t},m_{1,t},m_{2,t},\cdots ,m_{K,t},\gamma_{1,t},\gamma_{2,t},\cdots ,\gamma_{K,t}\right\}$$
  where $\left[\Delta_{1,t},\Delta_{2,t},\cdots ,\Delta_{K,t}\right]$ is the vector of length *K* containing the age of the oldest data packet in each UE, $\left[m_{1,t},m_{2,t},\cdots ,m_{K,t}\right]$ is the vector of length *K* containing the size of the oldest data packet in each UE, and $\left[\gamma_{1,t},\gamma_{2,t},\cdots ,\gamma_{K,t}\right]$ is the vector of length *K* containing the real-time SNR of the channels.
- Action Space: From (15), we use a vector of binary decision variables to determine whether the K-th UE transmits in frame *t*:
  (18) $$a_{t}=\left\{x_{1,t},x_{2,t},\cdots ,x_{K,t}\right\},x_{K,t}\in \left\{0,1\right\}$$
  where $x_{K,t}=1$ means UE K transmits a data packet to an edge server in frame *t*, and $x_{K,t}=0$ means UE K does not transmit a data packet in frame *t*. Since each *x* can be either 0 or 1, there are a total of $2^{n}$ possible actions for $a_{t}$.
- Reward Function: To minimize the average task AuD, the reward function we have designed is as follows:
  (19) $$r_{t}=-\alpha \Delta_{d,t}+C$$
  where $\Delta_{d,t}$ is the task AuD at frame *t*, α is a scaling factor that adjusts the sensitivity to changes in AuD, ensuring that the reward is neither too large nor too small, and *C* is a constant term that provides a fixed uplift to the reward, potentially aiding in learning stability and convergence of the learning process.

### 3.3. EMPPO Algorithm

EMPPO comes from the standard PPO algorithm and its adapted version maskable PPO. The PPO algorithm was proposed by John Schulman in 2017 [35] and has been widely adopted due to its simplicity, stability and efficiency. It is a policy gradient method, which provides an improvement to trust region policy optimization [36]. Then, considering scenarios where actions cannot always be selected, the maskable PPO has been proposed in 2022 [37]. By masking nonviable actions, it reduces ineffective exploration by agents, thereby enhancing the efficiency and performance of the algorithm.

**Figure 4:**
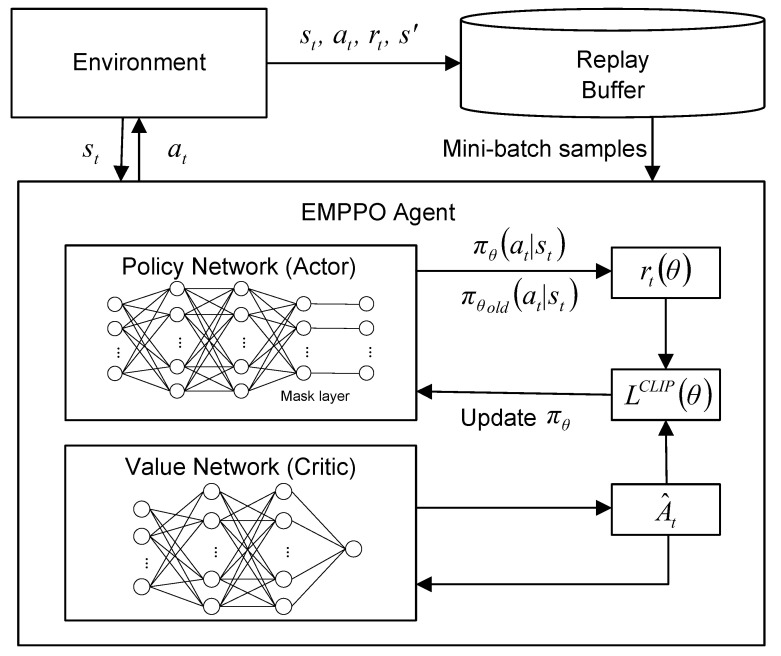
EMPPO framework.

The framework of EMPPO is shown in Figure 4. Within the EMPPO framework, action masking is critical in addressing the reliability constraint of low-latency MEC networks. Two main enhancements reflect EMPPO’s key advancements over the standard PPO. Firstly, EMPPO integrates a dynamic mask layer before the policy network’s output to evaluate and filter potential actions based on current conditions and ensure compliance with error probability constraints arising from variable CSI and packet sizes. Secondly, during forward propagation, EMPPO incorporates an additional step where the mask is actively applied to refine the action probability distribution, ensuring that only feasible actions are considered in decision-making processes.

In practice, EMPPO converts binary-encoded action vectors into one-hot encoded vectors, representing a unique mapping for all potential UE selections. Under the FBL regime, the mask function comes into play by selectively filtering actions according to real-time CSI.

EMPPO uses a clipped surrogate objective function to achieve a balance between exploitation and exploration, which can be expressed as:

(20) $$L^{CLIP}\left(\theta \right)={\hat{E}}_{t}\left[\min\left(r_{t}\left(\theta \right){\hat{A}}_{t},\mathrm{clip}\left(r_{t}\left(\theta \right),1-\epsilon ,1+\epsilon \right){\hat{A}}_{t}\right)\right]$$

where θ is the policy network parameter, $r_{t}\left(\theta \right)$ is the probability radio of action, ${\hat{A}}_{t}$ is the advantage estimate at timestep *t*, and ϵ is a hyperparameter.

The probability radio $r_{t}\left(\theta \right)$ can be denoted as:

(21) $$r_{t}\left(\theta \right)=\frac{\pi_{\theta }\left(a_{t}|s_{t}\right)}{\pi_{\theta_{old}}\left(a_{t}|s_{t}\right)}$$

where $\pi_{\theta }\left(a_{t}|s_{t}\right)$ is the current policy, $\pi_{\theta_{old}}\left(a_{t}|s_{t}\right)$ is the old policy. If $r_{t}\left(\theta \right)>1$, the agent is more likely to take action based on the current policy. If $0<r_{t}\left(\theta \right)<1$, the agent is more likely to take action based on the old policy.

The value network predicts the expected return from a given state, and the policy network outputs a probability distribution over actions given the current state. Generalized Advantage Estimation (GAE) is a method often used in PPO to reduce the variance of advantage estimates without greatly increasing bias, improving the stability of policy updates. The advantage function ${\hat{A}}_{t}$ can be calculated as:

(22) $${\hat{A}}_{t}=\delta_{t}+\left(\gamma \lambda \right)\delta_{t+1}+\cdots +{\left(\gamma \lambda \right)}^{T-t+1}\delta_{T-1}$$

where $r_{t}$ is reward, γ is the discount factor for future rewards and λ is GAE parameter. The temporal difference errors $\delta_{t}$ can be calculated as:

(23) $$\delta_{t}=r_{t}+\gamma V_{\phi }\left(s_{t+1}\right)-V_{\phi }\left(s_{t}\right)$$

where $V_{\phi }\left(s_{t+1}\right)$ is the value of the subsequent state, $V_{\phi }\left(s_{t}\right)$ is the value of the current state.

We employ an experience buffer for optimization. The agent’s experience is stored as tuples $\left(s,a,r,s^{\prime }\right)$ in an experience buffer and is then processed in epochs using mini-batches to improve policy parameters iteratively.

Based on the discussion, we can deploy the EMPPO algorithm in low-latency MEC environments. The specific steps are shown in Algorithm 1.
**Algorithm 1** EMPPO algorithm for UE selection in low-latency MEC network.**Require:** Initialize policy network $\pi_{\theta }\left(a|s\right)$ with parameters θ**Require:** Initialize value function $V_{\phi }\left(s\right)$ with parameters ϕ
**Require:** Initial policy parameters $\theta_{old}\leftarrow \theta$
**Require:** Hyperparameters: clipping parameter ϵ, discount factor γ, GAE parameter λ
1:Initialize experience replay buffer B2:**for** iteration←1 to *N* **do**3:    Collect a set of partial trajectories $D_{iteration}$ by running policy $\pi_{\theta_{old}}$4:    **for** each timestep *t* within these trajectories **do**5:        Compute advantage estimates ${\hat{A}}_{t}$ using GAE6:        Store transition $\left(s_{t},a_{t},r_{t},s^{\prime }\right)$ in buffer B7:    **end for**8:    **for** each epoch **do**9:         Sample mini-batch from buffer B10:       **for** each sampled transition $\left(s,a,r,s^{\prime }\right)$ **do**11:            Estimate probability ratio $r_{t}\left(\theta \right)$ using the current and old policies12:            Mask actions using function M(s)13:        **end for**14:        Optimize policy using the clipped objective function $L^{CLIP}\left(\theta \right)$15:        Update θ16:     **end for**17:     Update old policy parameters $\theta_{old}\leftarrow \theta$18:**end for**

## 4. Simulation Results

In this section, we evaluate the performance of the EMPPO algorithm within a simulated low-latency MEC environment, encompassing diverse scenarios such as random task arrivals, varying data packet sizes, and dynamic channel conditions. This assessment aims to show the adaptability of EMPPO in real-time offloading decisions against the baseline strategies.

We developed our low-latency MEC network simulation environment in Python 3.9 and refined the algorithms from the widely recognized open-source Stable Baselines3 reinforcement learning library [38] to formulate our EMPPO approach. The simulation experiments were conducted on a setup running Windows 11, comprising an AMD Ryzen 7 5800 CPU paired with 32 GB of system memory and an NVIDIA GeForce RTX 3070 Ti graphics card.

In particular, we consider a low-latency MEC network composed of three UEs and one edge server. The setting of specific parameters is based on the 3GPP protocol as much as possible to be close to the real scenario. The error probability constraint for packet transmission $\varepsilon_{max}=0.001$. Tasks arrive randomly in each frame with a probability p=0.4, and the size of the data packet $m_{k,i}$ arriving at each UE typically varies between 500 and 2500 bits. Each UE transmits data packets to the edge server through independent wireless channels. The average SNR of three channels may vary and are set as $\left[\bar{\gamma }-\Delta_{\bar{\gamma }},\bar{\gamma },\bar{\gamma }+\Delta_{\bar{\gamma }}\right]$, where $\bar{\gamma }=10$ dB, $\Delta_{\bar{\gamma }}=3$ dB. The channel correlation coefficient ρ is set as 0.8. The length of each frame is 1 ms, comprising 1500 symbols, with 1000 symbols for transmission and 500 symbols for computation, and one symbol’s duration is 66.7 μs.

In our proposed EMPPO framework, there are two networks: An actor network and a critic network. Both have one input layer, two hidden layers, and one output layer. The hidden layers are made up of 64 neurons. The other parameter settings of our algorithm are shown in Table 1.

To evaluate the proposed algorithm, we compare our algorithm with the following baselines.

(1) Random Offloading (RO): Randomly select some UEs for data packet offloading.
(2) Average Offloading (AO): Select UEs for data packet offloading according to a predefined sequence.
(3) Proximal Policy Optimization (PPO): The standard PPO algorithm, which has the same parameters as EMPPO.
(4) Verified Random Offloading (VRO): Randomly select some UEs to offload data packets and verify that all selected packets within a frame can meet the error probability constraints.
(5) Freshness Greedy Offloading (FGO): Always select the UE with the oldest packet for transmission, until the remaining blocklength makes the selected packet unable to meet the error probability constraints.
(6) Channel Greedy Offloading (CGO): Always select the UE with best channel state for transmission, until the remaining blocklength makes the selected packet unable to meet the error probability constraints.

To minimize the impact of randomness, our proposed method and all baselines will be tested 500 times, and the average result of these tests will be taken as the output.

**Figure 5:**
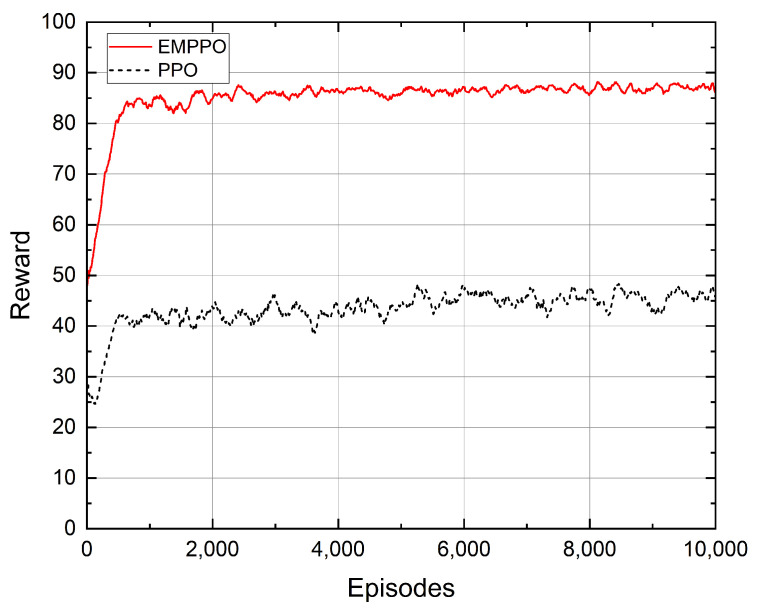
The convergence of reward.

Figure 5 shows the convergence performance of the proposed EMPPO and the standard PPO. EMPPO starts its learning process with an average reward of around 50, higher than PPO’s starting point of around 38. This is because EMPPO masks actions that can not meet the error probability constraints. Therefore, even when the action selection tends to be random at the beginning of the iteration, EMPPO can still achieve a better reward.

As the iterations progress, both EMPPO and PPO quickly improve their rewards in the first 500 episodes. After 500 episodes, the reward of EMPPO gradually converges to around 87, while the reward of PPO converges to about 45. This shows that EMPPO has a similar iteration efficacy to the standard PPO and can obtain a higher reward.

**Figure 6:**
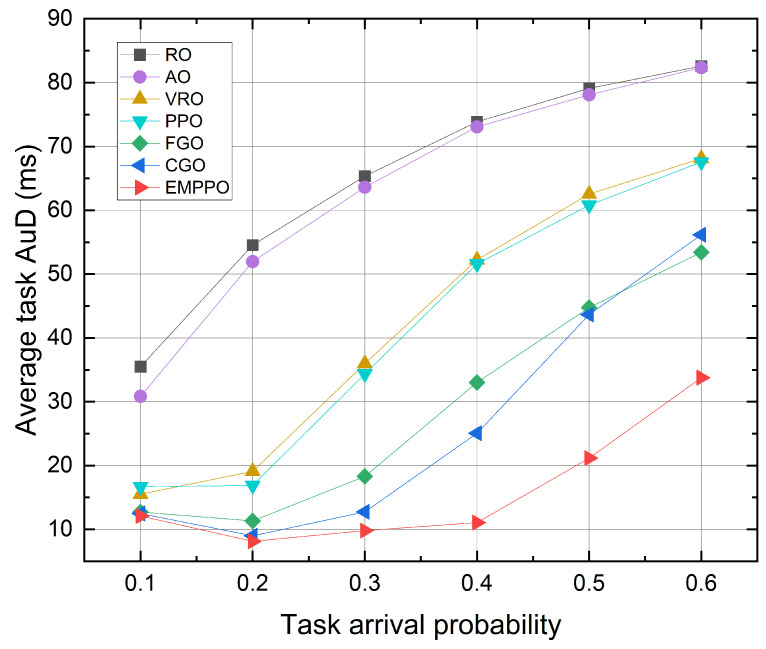
The average task AuD under different task arrival probability.

Figure 6 shows the influence of the task arrival probability. It can be seen that EMPPO has the lowest average task AuD across all task arrival probabilities, followed by the greedy methods FGO and CGO, followed by PPO, with RO constantly being the worst. As the task arrival probability increases, the average task AuD for all methods increases. This is due to an increased task arrival probability, potentially causing data packets to wait in the queue longer before being transmitted, and tasks have to wait longer for all the data packets to arrive. In this case, the average task AuD for CGO exceeds FGO’s, which means freshness-based packet transmission (i.e., FGO) is more efficient than channel-based transmission (i.e., CGO). This is because CGO tends to send all the packets from channels with good channel quality first, making it harder to transmit packets from channels with poor channel quality. There is a certain gap between the performance of PPO and EMPPO, and the gap gradually increases with the increase in task arrival probability. This indicates that EMPPO can deal with a large number of data packets more effectively than PPO, which may be because EMPPO reduces the exploration of invalid action space through variable masks.

At low task arrival probability, EMPPO’s performance is comparable to that of FGO and CGO, and the average task AuD initially decreases and then increases as the probability rises. This can be attributed to the less frequent arrival of new tasks and, as a result, a decelerated refresh rate of the task AuD. At high task arrival probability, EMPPO significantly reduces the average task AuD more than other methods. This suggests that EMPPO can successfully balance packet freshness and channel conditions, performing better under high-load scenarios.

**Figure 7:**
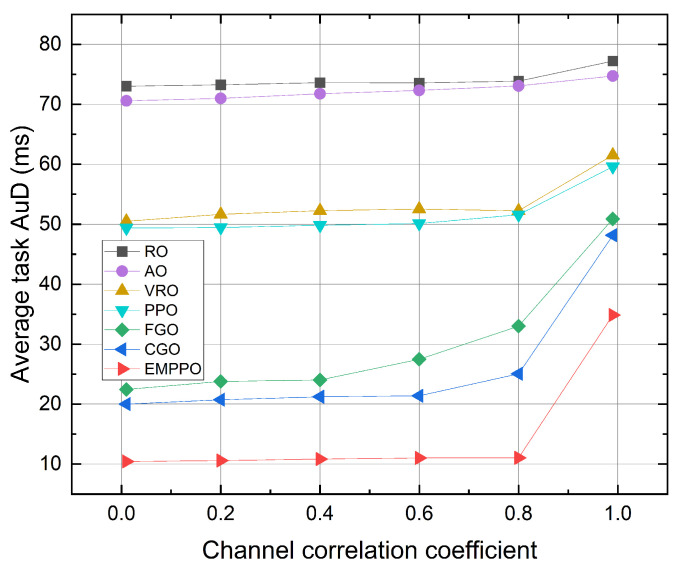
The average task AuD under different channel correlation coefficients.

Figure 7 shows the influence of the channel correlation coefficient. EMPPO consistently achieves the lowest average task AuD through various channel correlation coefficients. The average task AuD time for all methods exhibits a slow increase following a rise in the channel correlation coefficient. Among these, the greedy methods FGO and CGO show a greater sensitivity to changes in the channel correlation coefficient. A significant increase in average task arrival time is observed under the extreme channel correlation coefficient ρ=0.99. This is because the channel conditions change slowly at this channel correlation coefficient, making it difficult to take advantage of channel variations and transmit data packets with shorter blocklength during optimal channel conditions.

**Figure 8:**
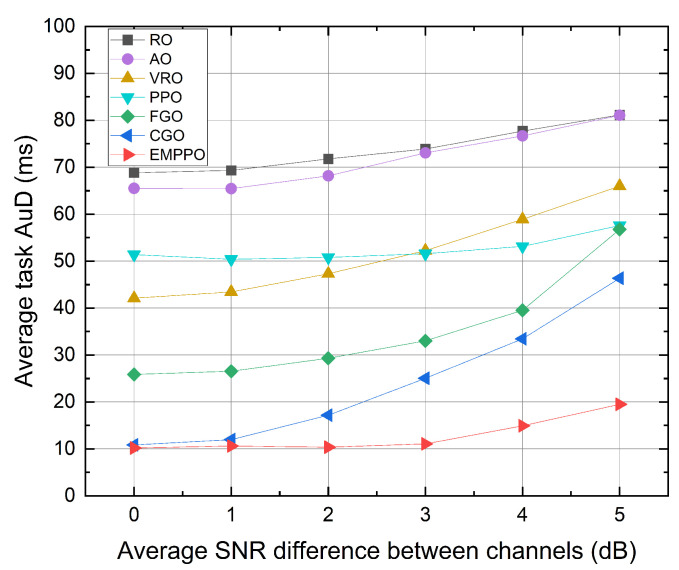
The average task AuD under different average SNR differences between channels.

Figure 8 shows the influence of the average SNR difference between channels. As the difference increases, the average task AuD for all methods grows, with EMPPO recording the lowest average task AuD, followed by CGO. Also, the gap in average task AuD between EMPPO and CGO gradually increases. This is mainly because CGO selects the UE corresponding to the channel with the highest SNR for data transmission. When the difference is zero, the channels can be regarded as three independent channels with the same average SNR, having equal transmission probabilities for each UE. However, as the difference increases, the channel with the highest average SNR is more likely to transmit data, while the channel with the lowest average SNR is likely to accumulate data packets. Figure 8 also shows that PPO and EMPPO methods based on reinforcement learning are less affected by the increase in channel differences, indicating that the method based on reinforcement learning can adapt to and balance the transmission of channels under different channel states.

**Figure 9:**
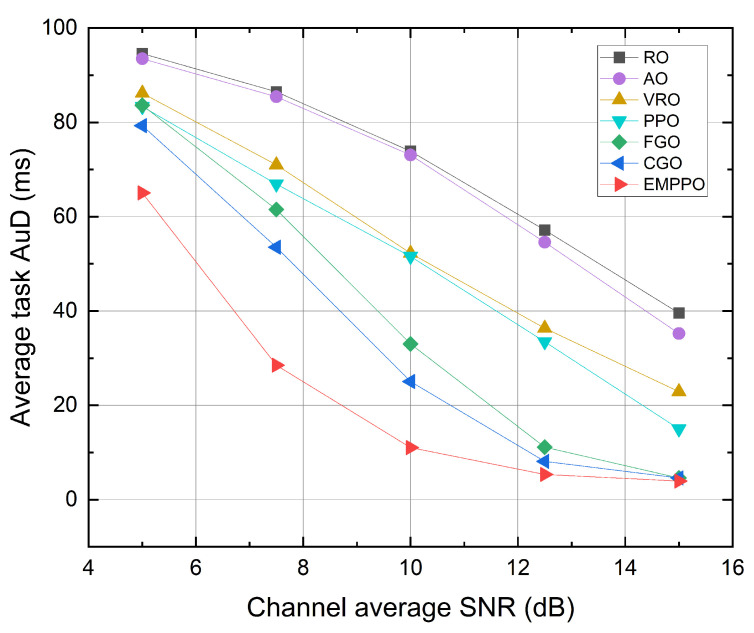
The average task AuD under different channel average SNR.

Figure 9 shows the influence of the channel average SNR. As the SNR increases, the average task AuD of each method decreases. When the average SNR is high, data packets can be transmitted with a shorter blocklength, allowing more packets to be transmitted in a single frame. The results of methods based on greedy algorithms tend to be closer to EMPPO. However, when the average SNR drops, data packets require longer blocklength for transmission, and often, a task’s data packets could be transmitted over multiple frames. In this scenario, deciding which data packets are transmitted in which frames significantly impacts the average task AuD. EMPPO performs better than other methods, suggesting that EMPPO is more applicable in environments with a low SNR. In extremely low SNR environments, a frame’s total blocklength might not be enough to transmit a single data packet. Thus, the average task AuD differences among different methods become less pronounced.

**Figure 10:**
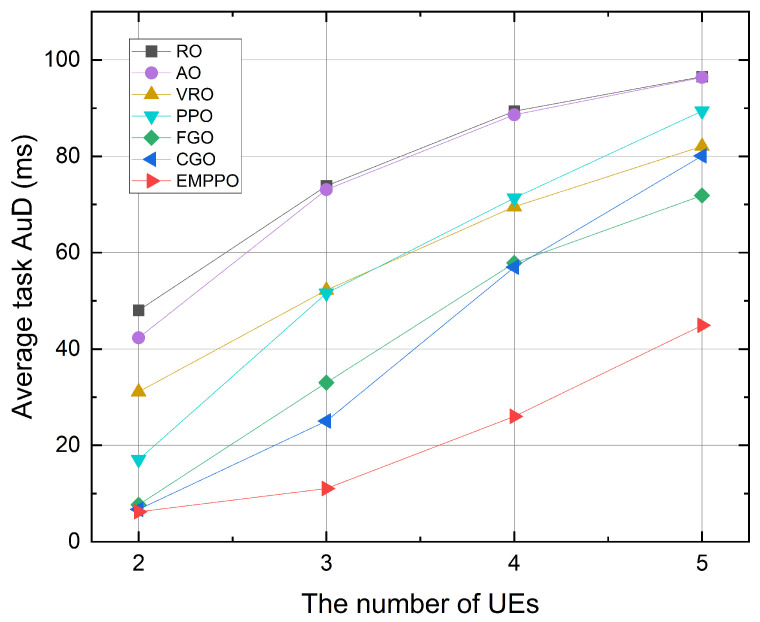
The average task AuD under a different number of UEs.

In Figure 9, we can observe that RO and AO show different convexity characteristics from other methods. That is because their performance approaches the maximum average task AuD. The maximum average task AuD is influenced by the number of simulation steps, which is set to 200 in the simulation. This leads to a maximum average task AuD of 100 ms (this corresponds to scenarios where no data packets are transmitted in any frame). An algorithm performing close to this upper bound will show a reduced rate of change, hence showing the distinct convexity. We can also observe this trend in Figure 6 and Figure 10.

Figure 10 shows the influence of UE number. When the number of UEs increases, the task AuD of each method increases. This is because the one task’s packet number is increasing (equal to the number of UEs). While the available blocklength for transmission is fixed, we need more frames to transmit the packets for one task. When the number of UEs is small, EMPPO’s task AuD is similar to that of FGO and CGO. However, when the number of UEs is large, the performance of EMPPO is significantly better than other methods, which is consistent with the performance under different task arrival probabilities and confirms our analysis of Figure 6; that is, EMPPO can achieve better performance than other methods under high-load scenarios, and standard PPO has a lot of invalid action space to explore, which leads to a lower task AuD.

**Figure 11:**
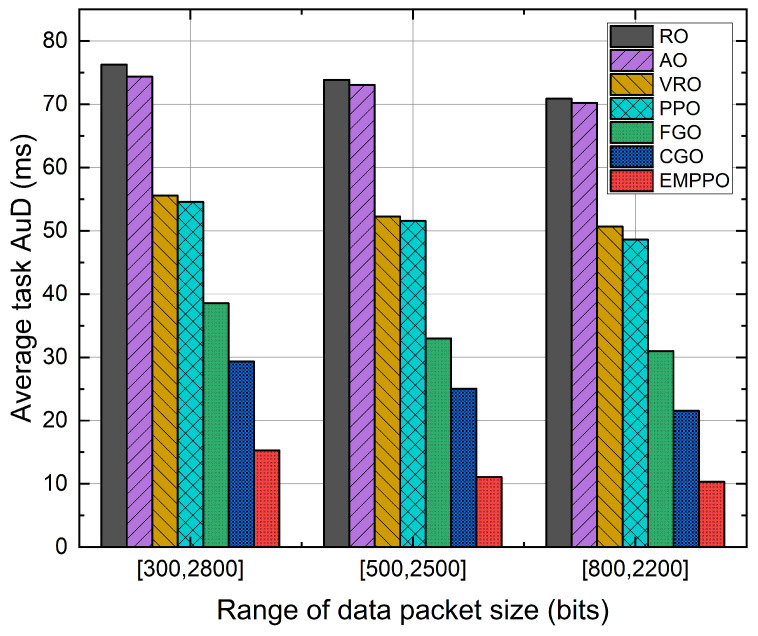
The average task AuD under different ranges of data packet size.

Figure 11 shows the influence of data packet size range. We have set up three different ranges for data packet size, each with the same average packet size of m=1500 bits. Figure 11 shows that the average task AuD decreases as the packet size range decreases. Specifically, when the range is set to [800,2200], the average task AuD of EMPPO drops by about 33% and 7% compared to ranges [300,2800] and [500,2500]. On the one hand, this is because a smaller packet size range results in a more concentrated distribution of packet size, which leads to a more focused distribution of the required blocklength, allowing the agent to predict the required blocklength for packets more accurately. On the other hand, the probability of encountering a large packet size is reduced, which decreases the probability of packets requiring a blocklength that exceeds frame length constraints. This enables packets to be transmitted successfully under relatively poor channel SNR.

## 5. Conclusions

In this work, we proposed a latency optimization design for low-latency MEC networks. We optimized the average AuD for tasks through UEs selection and blocklength allocation. Specifically, we considered random fading channels and packet transmission with FBL codes. We formulated an optimization problem to minimize the average task AuD under error probability constraints and, based on the FBL regime, transformed the joint optimization problem into an effective UE selection problem. Due to the dynamic task generation and random fading channels, the problem cannot be well solved analytically. We further proposed a DRL approach with the action-masked PPO method, which dynamically masks actions that cannot satisfy error probability constraints.

Simulation results validated the effectiveness of our proposed EMPPO method. In particular, our method avoids exploring a large volume of the ineffective action space through the mask, significantly outperforming standard PPO methods. It also adapts better to scenarios with poor channel SNR and heavy task loads, reflecting the adaptability of DRL-based methods to dynamic environments. It is also worth mentioning that, although we assumed real-time CSI can be obtained, the proposed design can be extended to scenarios where only outdated or imprecise CSI is available, which is more realistic. Under such conditions, strict error probability constraints cannot be enforced, representing an exciting direction for our future work.

## Figures and Tables

**Table 1:** EMPPO algorithm parameters.

Description	Symbol	Value
Number of iterations	*N*	$1\times 10^{6}$
Number of episodes	$n_{e}$	5000
Learning Rate	α	0.0003
Number of Steps	$n_{steps}$	2000
Batch Size	*B*	100
Discount Factor	γ	0.99
Clip Range	ρ	0.2
Exploration Rate	ϵ	1
Exploration Decay Rate	δ	0.995
GAE Factor	$\lambda^{GAE}$	0.95

## Data Availability

All data generated or analyzed during this study are given in Section 4.

## Abbreviations

The following abbreviations are used in this manuscript:

MEC	Mobile Edge Computing
FBL	Finite Blocklength
UE	User Equipment
AoI	Age of Information
AuD	Age upon Decision
PPO	Proximal Policy Optimization
IoT	Internet of Things
URLLC	Ultra-Reliable Low-Latency Communications
MDP	Markov Decision Process
SNR	Signal-to-Noise Ratio
FIFO	First-In-First-Out
GAE	Generalized Advantage Estimation
EMPPO	Error Probability-Controlled Action-Masked Proximal Policy Optimization
